# Regression Models to Study the Total LOS Related to Valvuloplasty

**DOI:** 10.3390/ijerph19053117

**Published:** 2022-03-07

**Authors:** Arianna Scala, Teresa Angela Trunfio, Lucia De Coppi, Giovanni Rossi, Anna Borrelli, Maria Triassi, Giovanni Improta

**Affiliations:** 1 Department of Public Health, University of Naples “Federico II”, 80131 Naples, Italy; ariannascala7@gmail.com (A.S.); luciadecoppi@gmail.com (L.D.C.); triassi@unina.it (M.T.); ing.improta@gmail.com (G.I.); 2 Department of Advanced Biomedical Sciences, University of Naples ‘Federico II’, 80131 Naples, Italy; 3 Hospital Directorate, “San Giovanni di Dio e Ruggi d’Aragona” University Hospital of Salerno, 84125 Salerno, Italy; giovanni.rossi@sangiovannieruggi.it (G.R.); acquarama@libero.it (A.B.); 4 Interdepartmental Center for Research in Healthcare Management and Innovation in Healthcare (CIRMIS), University of Naples “Federico II”, 80131 Naples, Italy

**Keywords:** valvuloplasty, length of stay, regression

## Abstract

**Background:** Valvular heart diseases are diseases that affect the valves by altering the normal circulation of blood within the heart. In recent years, the use of valvuloplasty has become recurrent due to the increase in calcific valve disease, which usually occurs in the elderly, and mitral valve regurgitation. For this reason, it is critical to be able to best manage the patient undergoing this surgery. To accomplish this, the length of stay (LOS) is used as a quality indicator. **Methods:** A multiple linear regression model and four other regression algorithms were used to study the total LOS function of a set of independent variables related to the clinical and demographic characteristics of patients. The study was conducted at the University Hospital “San Giovanni di Dio e Ruggi d’Aragona” of Salerno (Italy) in the years 2010–2020. **Results:** Overall, the MLR model proved to be the best, with an R^2^ value of 0.720. Among the independent variables, age, pre-operative LOS, congestive heart failure, and peripheral vascular disease were those that mainly influenced the output value. **Conclusions:** LOS proves, once again, to be a strategic indicator for hospital resource management, and simple linear regression models have shown excellent results to analyze it.

## 1. Introduction

The present research paper is an extension of a previous paper that the same authors presented at a conference [1]. In fact, the dataset considered is much larger, both in terms of number of records and the variables considered. Moreover, in order to further improve the regression model, a comparison with other algorithms was made. 

Valvular heart diseases are diseases that affect the valves by altering the normal circulation of blood within the heart, with repercussions on the general health of the subject.

Knowledge of the natural history of the most common valvular heart diseases is important because the onset of symptoms often is the point at which intervention becomes necessary. Most valvular heart diseases are amenable to surgical intervention, which can afford a symptom-free and relatively normal life span [2].

The prevalence of valvular disease increases sharply with age, owing to the predominance of degenerative etiologies. The burden of heart valve disease in the elderly has an important impact on patient management, given the high frequency of comorbidity and the increased risk associated with intervention in this age group [3].

For each subject, it is fundamental to evaluate the severity of valvular disease, given that the risk in surgery is proportional to the degree of valvular disease; specifically in the elderly, any type of surgery pre-operative evaluation and preparation is especially important for a successful outcome of the surgery [4].

A prospective survey of patients with valvular heart disease in Europe showed that of patients with severe, symptomatic, single VHD, 31.8% did not undergo intervention, most frequently because of comorbidities [5].

The etiology, approach to treatment, and expected outcomes of VHD are different in the elderly compared with younger patients. Both stenotic and regurgitant lesions are associated with unfavorable outcomes if left untreated. Surgical mortality remains high due to multiple co-morbidities, and the long-term survival benefit is dependent on many variables, including valvular pathology. Quality of life is an important consideration in treatment decisions in this age group. Increasingly, octogenarian patients are receiving transcatheter therapies, with transcatheter aortic valve replacement having the greatest momentum [6].

When surgery is not possible, or when the risks outweigh the benefits, percutaneous treatment options may offer effective alternatives. However, procedures may not always go as planned, and frail patients or those whose symptoms are caused by other comorbidities may not benefit from valve intervention at all. Significant effort should be made to assess frailty, comorbidities, and patient goals prior to intervention [7].

In the current guidelines of the European Society for Cardiology, published in 2021 [8], surgical treatment remains the standard of care for most forms of severe valvular heart disease; however, the presence of chronic kidney disease impairs clinical outcomes and is associated with higher mortality rates when compared to patients with preserved renal function [9].

These latter valvular abnormalities are likely to increase further as the average age of the population increases [10]. For this reason, it is critical to be able to best manage the patient undergoing this surgery.

The length of hospital stay (LOS) is considered an excellent indicator of quality in care processes [11,12]. In fact, many studies have focused on how to reduce patients’ LOS by optimizing care processes. For example, Scala et al. and Improta et al. demonstrated how the introduction of a diagnostic therapeutic pathway for femur fracture and diabetic patient management, respectively, reduced LOS with consequent benefits for both patients and hospitals [13,14]. Biomedical data analytics is key to improving processes, reducing costs, and giving clinicians new tools to manage all different patients. There are many approaches used in the literature for data analysis, including lean six sigma [15,16,17,18,19], health technology assessment [20,21,22,23], machine learning algorithms [24,25,26], and mathematical modelling [27,28,29]. The latter approach was chosen for this study. In the literature, there are several applications: Tesfahun et al., in order to optimize medical waste management processes, developed a model capable of predicting the production rate of this waste [30]; Chatterjee et al. obtained a model capable of predicting the spread of viruses [31]; Liu et al. employed a regression to evaluate the clinical factors that most influence LOS in adult patients with peritonsillar abscess [32]; and Kadam et al. employed both artificial neural networks and a multiple linear regression to predict the potability of water in an Indian river [33].

Therefore, the aim of this work is to use mathematical modelling and, in particular, several regression algorithms to obtain a model that can help clinicians in the assessment of the LOS of patients undergoing mitral valve repair surgery. As already mentioned, the present work aims to be an extension and improvement of the previous one presented at a conference [1]. In fact, the final model is more complex since the individual comorbidities were considered and, therefore, allows the clinician to take into account more aspects that characterize the particular patient.

## 2. Method

The research was carried out at the Complex Operative Unit (C.O.U.) of the Cardiology unit at the University Hospital “San Giovanni di Dio e Ruggi d’Aragona” of Salerno (Italy). The dataset was obtained from the hospital’s information system, QuaniSDO, and included all patients who underwent open-heart mitral valve repair surgery without replacement from 2010 to 2020. It comprises 379 records and contains the following information:

- Gender (male/female);
- Age;
- Comorbidities;
- Diagnostic-related group (DRG);
- Procedures;
- Date of admission, discharge, and procedure.

From this information, variables were obtained that were then used in the multiple linear regression. In particular, the dependent variable (i.e., the output of the model), the LOS, was obtained as the difference between the date of discharge and the date of admission; the pre-operative LOS-independent variable was obtained as the difference between the date of discharge and the date of the procedure. By analyzing the procedures, it was possible to determine the number of cardiac procedures carried out in addition to mitral valve repair surgery. For example, interventions for bypass, pacemaker implantation, cardioversions, or other interventions on valves were considered. In addition, the other independent variables are reported below:

- Gender (male/female);
- Age;
- Acute myocardial infarction (AMI) (yes/no);
- Congestive heart failure (CHF) (yes/no);
- Cerebrovascular disease (CeVD) (yes/no);
- Peripheral vascular disease (PVD) (yes/no);
- Chronic obstructive pulmonary disease (COPD) (yes/no);
- Diabetes (yes/no);
- Renal disease (RD) (yes/no).
- Two procedures;
- Three procedures;
- Four procedures.

Table 1 shows the distribution of the features into the sample.

### Regression Algorithms

IBM SPSS (Statistical Package for Social Science, IBM Corporation, Armonk, NY, USA) ver. 27 [34] was used to build an MLR model used to predict the total LOS. Before its implementation, the following six conditions must be verified:

1. Linear relationship between the independent and dependent variable;
2. Absence of collinearity;
3. Independence of the residuals;
4. Constant variance of the residuals;
5. Normal distribution of residuals;
6. Absence of outliers.

With MatLab version R2020a, other regression algorithms (linear support vector machine, LSVM; narrow neural network, NNN; rational quadratic Gaussian process regression, GPR; and random forest, RF) were implemented. In particular, SVM can also be used as a regression method, keeping at the base the same main idea of the classifier to minimize the error by identifying a hyperplane in an N-dimensional space, where N depends on the number of variables, and considering a margin of tolerance that is not part of the classification process. Neural network models can be considered valid alternatives to classical regression models. In fact, they have the property of learning from a set of data without the need for a complete specification of the decision model. They automatically provide all necessary data transformations and are able to see through noise and distortion. Gaussian processes (GP) are a supervised learning method used for regression and probabilistic classification problems. They are versatile, different kernels can be specified, and the prediction is probabilistic (Gaussian). Lastly, RF is a supervised learning algorithm in which multiple learning algorithms are combined together to make a more accurate prediction. The model is powerful and accurate, but overfitting can easily occur. Before performing the analyses, the dataset was divided into training sets for 80% and test sets for 20%. The R^2^ parameter was used to evaluate the accuracy of the model.

## 3. Results

Before implementing the MLR model, the six hypotheses were tested.

### 3.1. The Linear Relationship between the Independent and Dependent Variable

**Figure 1:**
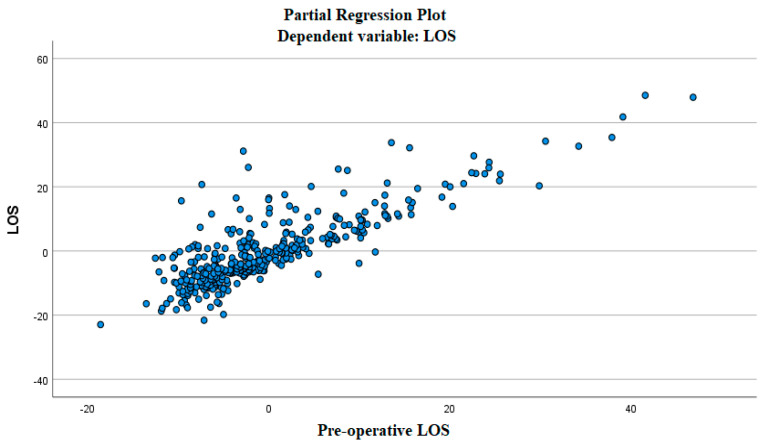
Partial regression plot (pre-operative LOS).

To verify this assumption, partial dispersion graphs were created to verify the trend of the dependent variable LOS as a function of the selected independent variables. Figure 1 shows what has been obtained for the pre-operative LOS.

Consistent with the definition of total LOS, the linear relationship between the variables was deduced. The problem with this type of representation was that the effect of combining several independent variables was not considered.

### 3.2. Absence of Multicollinearity

The absence of multicollinearity has been demonstrated through Pearson’s correlation, tolerance, and the variance inflation factor (VIF). All variables are a function of the correlation between the i-th independent variable and the others. Table 2 shows the results of the Pearson correlation and the statistical significance.

Table 3, instead, shows the values of VIF and tolerance that were obtained for each independent variable.

With the exception of the pre-operative LOS, the Pearson correlation value was always less than 0.7. In addition, the VIF values were always less than 10 and the tolerance values were always greater than 0.2, so the absence of multicollinearity was verified.

### 3.3. The Independence of the Residuals

The Durbin–Watson statistical test was used to test this hypothesis. The result is always between 0 and 4, where the intermediate value represents that there is no autocorrelation detected in the sample. In this case, the result was equal to 1.517 and, therefore, was within the acceptability range of (1.5; 2.5).

### 3.4. The Residuals Have Constant Variance

**Figure 2:**
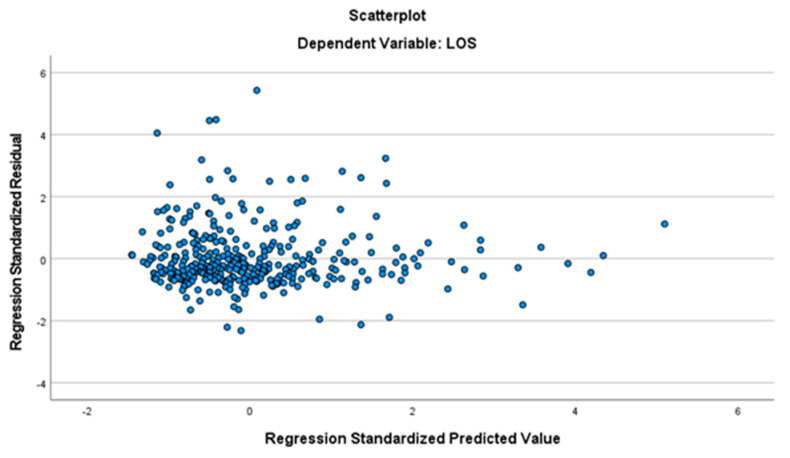
Homoscedasticity of the data.

To evaluate the variance of the residuals, the graphic “standardized expected value regression” on the x-axis against “standardized residual regression” was created. Figure 2 shows the obtained result.

The scatter plot (Figure 2) shows that the data is randomly distributed around zero. It is possible to say that the homoscedasticity hypothesis is not violated. The hypothesis is therefore verified.

### 3.5. The Residuals Are Normally Distributed 

**Figure 3:**
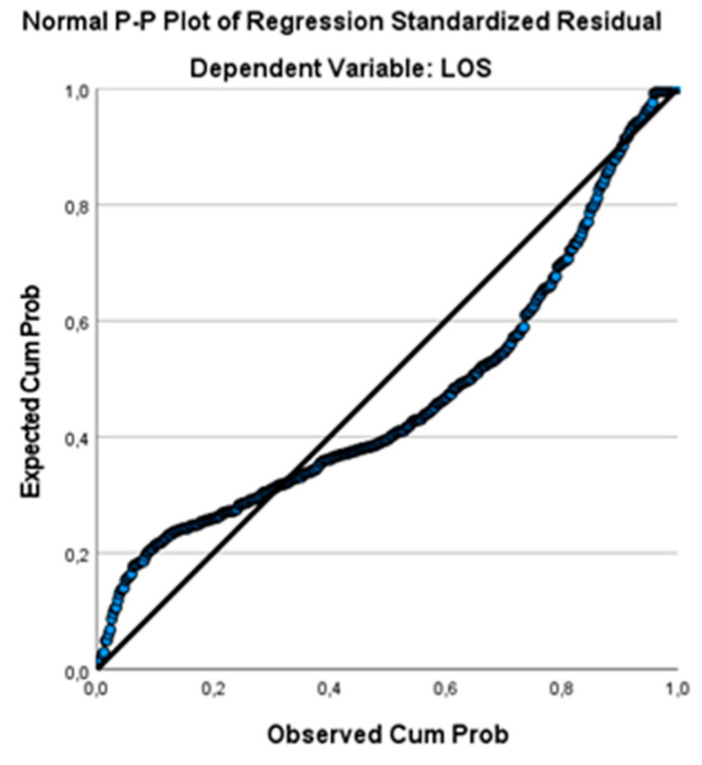
P–P plot.

The P–P plot (Figure 3) shows how well the available data set fits the specific probability distribution. With this tool, the cumulative distribution of the empirical probability of the data is compared with that of the assumed true cumulative distribution functions.

Although the curve did not exactly retrace the ideal line, the slight variation did not affect the good performance of the model.

### 3.6. Presence of Outliers

**Figure 4:**
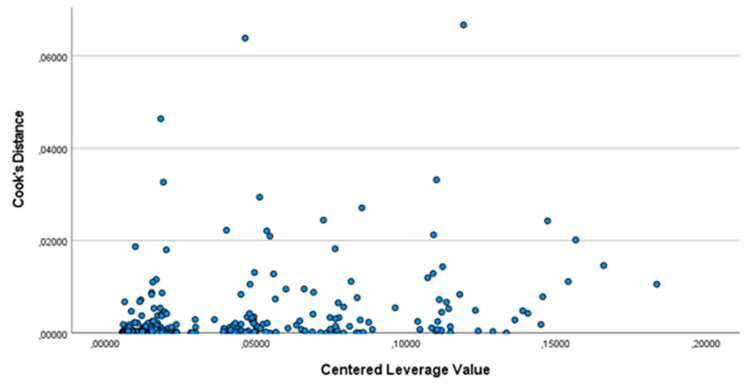
Cook’s distance.

The last hypothesis to be verified was the absence of outliers that affect the estimate of the parameters βi. To accomplish this, Cook’s distance was calculated for each observation. Figure 4 shows the obtained result.

For each observation, the Cook’s distance was less than 1. Therefore, there were no outliers that caused bias.

After this verification phase, the MLR model was implemented. Table 4 shows the goodness of the model.

The R^2^ value was greater than the set threshold value of 0.5. The model was well suited to the problem under consideration and could be a valid preliminary tool. Table 5 shows the model coefficients and the t-test result at a significance level of 0.05.

The test showed that of the selected independent variables, age, pre-operative LOS, CHF, and PVD significantly influenced LOS. For all of them, the value of the coefficients was positive and among these the highest was the one associated with the PVD.

After analyzing the results of the MLR model, further regression algorithms were implemented (Table 6).

**Figure 5:**
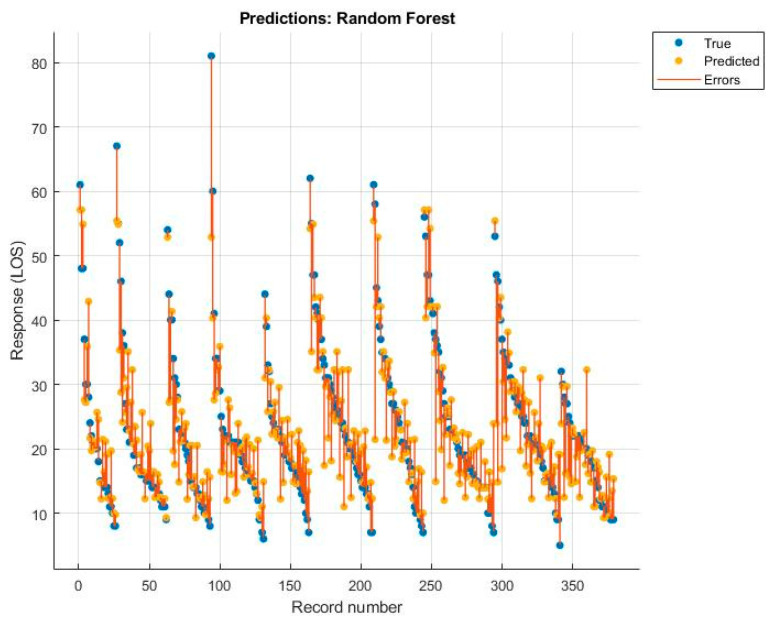
Random forest.

**Figure 6:**
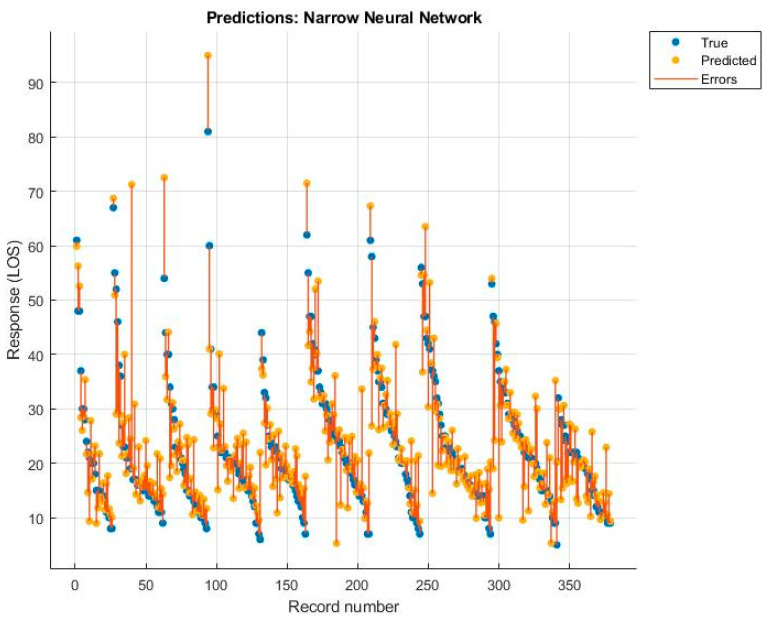
Narrow neural network.

**Figure 7:**
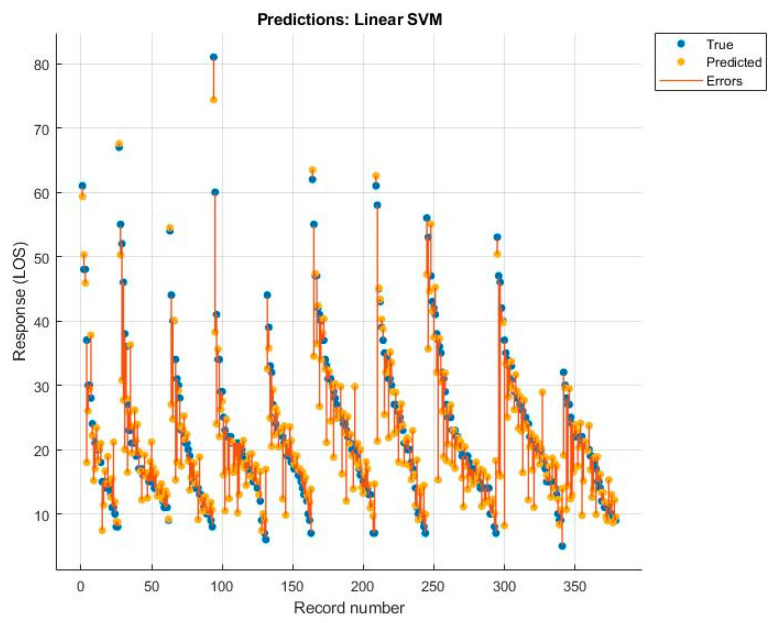
Linear support vector machine.

**Figure 8:**
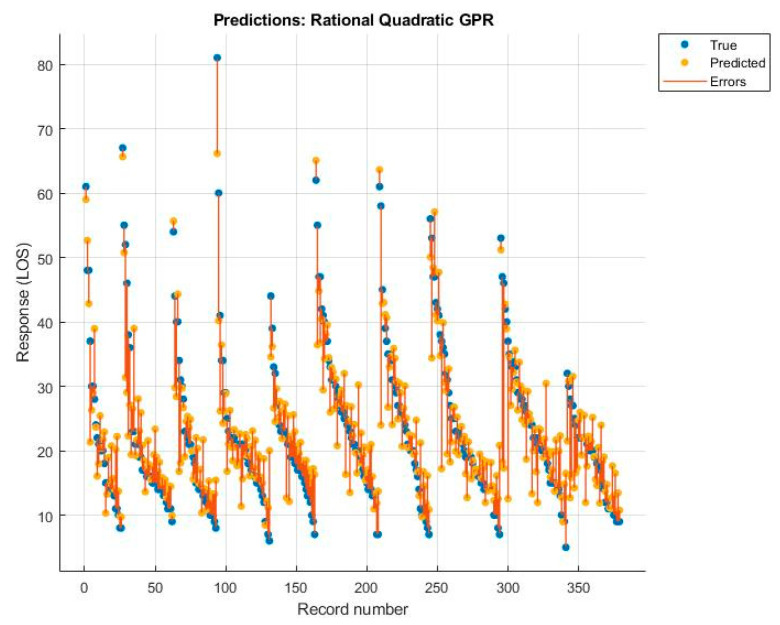
Rational quadratic GPR.

Among these, the best was the rational quadratic GPR, but the value was still lower than that obtained with the MLR model. The diagrams of the predictions made, with the relative errors for each algorithm, are shown below (Figure 5, Figure 6, Figure 7 and Figure 8).

## 4. Discussion

In this study, the data provided by the C.O.U. of the Cardiology unit at the University Hospital “San Giovanni di Dio e Ruggi d’Aragona” of Salerno (Italy) were analyzed. Specifically, the information was related to the flow of patients who underwent an open-heart mitral valve repair surgery without replacement from 2010 to 2020, for a total of 379 records. Starting from the extraction of a limited set of information from hospital discharge forms, a group of independent variables were obtained (gender, age, pre-operative LOS, acute myocardial infarction (AMI), congestive heart failure (CHF), cerebrovascular disease (CeVD), peripheral vascular disease (PVD), chronic obstructive pulmonary disease (COPD), diabetes, renal disease (RD), 2 procedures, 3 procedures and 4 procedures) and were used to predict the total LOS. As conducted in the previous study [1], an MLR model was implemented. The obtained MLR model had an R^2^ value equal to 0.720, and among the variables, those that most influenced the LOS were age, pre-operative LOS, CHF, and PVD. Compared to the result obtained in the short paper, where the model was obtained using 70 records included in the 379 used here, the goodness of the model is slightly lower (R^2^ = 0.864) without showing, with the exception of the pre-operative LOS, which is linked to the LOS by definition, any significant influence. Undergoing multiple heart surgeries was not significantly correlated with LOS. In this case, the greater number of records made it possible to identify the classes of patients for which greater organizational effort is required. In addition to the MLR model, further regression algorithms were tested (linear support vector machine, LSVM; narrow neural network, NNN; rational quadratic Gaussian process regression, GPR; and random forest, RF). Of these, the best was rational quadratic GPR, with a value of R^2^ = 0.690. The performance, however, was lower than the MLR model, which ultimately remains the best model.

The limitation of this work is certainly that of not considering the impact that specific cardiac procedures with the same complexity as the one in the exam, such as coronary revascularization and tricuspid annuloplasty, have on LOS.

Future developments will certainly include exceeding the limits, the validation of the models through both an update of the dataset with the inclusion of what has been obtained for the year 2021 and through the analysis of data from different populations. In addition, further regression and classification models may be implemented.

## 5. Conclusions

In this work, the dataset consisting of 379 patients who underwent open-heart mitral valve repair surgery without replacement from 2010 to 2020 at the C.O.U. of the Cardiology unit at the University Hospital “San Giovanni di Dio e Ruggi d’Aragona” of Salerno (Italy) was analyzed through 4 different regression models/algorithms. An MLR model, linear support vector machine, narrow neural network, rational quadratic Gaussian process regression, and random forest were implemented. Among these, the best was the MLR model, with an R^2^ = 0.720. Finally, the statistical analysis showed that the variables that significantly affected the total LOS were age, pre-operative LOS, congestive heart failure, and peripheral vascular disease.

## Figures and Tables

**Table 1:** Features of dataset.

Features	Dataset (N = 379)
Gender	
M	199
F	180
AMI	
Yes	13
No	366
CHF	
Yes	118
No	261
CeVD	
Yes	16
No	363
PVD	
Yes	11
No	368
COPD	
Yes	27
No	352
Diabetes	
Yes	17
No	362
RD	
Yes	27
No	352
2 Procedures	
Yes	135
No	244
3 Procedures	
Yes	33
No	347
4 Procedures	
Yes	11
No	370

**Table 2:** Pearson correlation and statistical significance.

Pearson Correlation	Variable/Variable	LOS	Age	Gender	Pre-operative LOS	AMI	CHF	PVD	CeVD	COPD	Diabetes	RD	2 procedures	3 procedures	4 procedures
	LOS	1.000	0.098	−0.031	0.829	−0.010	0.231	0.184	0.081	0.005	0.035	0.047	0.105	0.106	0.098
	Age	0.098	1.000	0.126	−0.034	−0.033	0.117	0.069	0.084	0.111	0.145	0.195	0.106	0.081	0.025
	Gender	−0.031	0.126	1.000	−0.048	−0.063	−0.035	−0.070	0.011	−0.140	−0.053	−0.079	−0.001	−0.013	−0.058
	Pre-operative LOS	0.829	−0.034	−0.048	1.000	0.026	0.143	0.117	0.064	−0.002	0.043	0.005	0.081	0.048	0.084
	AMI	−0.010	−0.033	−0.063	0.026	1.000	−0.001	−0.033	0.033	0.004	0.029	0.061	−0.110	−0.007	−0.031
	CHF	0.231	0.117	−0.035	0.143	−0.001	1.000	0.053	−0.056	0.057	0.019	0.035	0.154	0.055	0.103
	PVD	0.184	0.069	−0.070	0.117	−0.033	0.053	1.000	0.042	−0.048	−0.037	0.074	0.101	0.170	−0.028
	CeVD	0.081	0.084	0.011	0.064	0.033	−0.056	0.042	1.000	−0.058	0.145	0.095	−0.047	0.028	−0.035
	COPD	0.005	0.111	−0.140	−0.002	0.004	0.057	−0.048	−0.058	1.000	−0.010	0.003	−0.013	0.096	0.018
	Diabetes	0.035	0.145	−0.053	0.043	0.029	0.019	−0.037	0.145	−0.010	1.000	0.039	0.052	0.069	0.203
	RD	0.047	0.195	−0.079	0.005	0.061	0.035	0.074	0.095	0.003	0.039	1.000	−0.013	0.024	−0.046
	2 procedures	0.105	0.106	−0.001	0.081	−0.110	0.154	0.101	−0.047	−0.013	0.052	−0.013	1.000	0.415	0.221
	3 procedures	0.106	0.081	−0.013	0.048	−0.007	0.055	0.170	0.028	0.096	0.069	0.024	0.415	1.000	0.533
	4 procedures	0.098	0.025	−0.058	0.084	−0.031	0.0103	−0.028	−0.035	0.018	0.203	−0.046	0.221	0.533	1.000
Sign. (1-Tailed)	Variable/Variable	LOS	Age	Gender	Pre-operative LOS	AMI	CHF	PVD	CeVD	COPD	Diabetes	RD	2 procedures	3 procedures	4 procedures
	LOS	.	0.028	0.275	0.000	0.425	0.000	0.000	0.058	0.463	0.250	0.183	0.020	0.020	0.028
	Age	0.028	.	0.007	0.252	0.263	0.011	0.090	0.051	0.015	0.002	0.000	0.020	0.058	0.316
	Gender	0.275	0.007	.	0.177	0.110	0.250	0.087	0.419	0.003	0.152	0.063	0.490	0.403	0.131
	Pre-operative LOS	0.000	0.252	0.177	.	0.304	0.003	0.011	0.105	0.482	0.202	0.459	0.058	0.177	0.052
	AMI	0.425	0.263	0.110	0.304	.	0.488	0.264	0.264	0.468	0.285	0.120	0.016	0.448	0.274
	CHF	0.000	0.011	0.250	0.003	0.488	.	0.150	0.138	0.132	0.353	0.247	0.001	0.142	0.023
	PVD	0.000	0.090	0.087	0.011	0.264	0.150	.	0.208	0.176	0.234	0.074	0.025	0.000	0.290
	CeVD	0.058	0.051	0.419	0.105	0.264	0.138	0.208	.	0.129	0.002	0.032	0.183	0.292	0.251
	COPD	0.463	0.015	0.003	0.482	0.468	0.132	0.176	0.129	.	0.420	0.476	0.399	0.030	0.360
	Diabetes	0.250	0.002	0.152	0.202	0.285	0.353	0.234	0.002	0.420	.	0.224	0.157	0.091	0.000
	RD	0.183	0.000	0.063	0.459	0.120	0.247	0.074	0.032	0.476	0.224	.	0.399	0.323	0.188
	2 procedures	0.020	0.020	0.490	0.058	0.016	0.001	0.025	0.183	0.399	0.157	0.399	.	0.000	0.000
	3 procedures	0.020	0.058	0.403	0.177	0.448	0.142	0.000	0.292	0.030	0.091	0.323	0.000	.	0.000
	4 procedures	0.028	0.316	0.131	0.052	0.274	0.023	0.290	0.251	0.360	0.000	0.188	0.000	0.000	.

**Table 3:** Collinearity statistics.

Independent Variables	Tolerance	Variance Inflation Factor
Age	0.871	1.148
Gender	0.926	1.080
Pre-operative LOS	0.947	1.056
AMI	0.973	1.028
CHF	0.931	1.074
PVD	0.913	1.095
CeVD	0.943	1.060
COPD	0.932	1.073
Diabetes	0.907	1.102
RD	0.933	1.072
2 Procedures	0.783	1.277
3 Procedures	0.576	1.736
4 Procedures	0.652	1.534

**Table 4:** Model summary.

	R	R^2^	R^2^ Adjusted	Std. Error of the Estimate
MLR Model	0.850	0.722	0.712	6.331

**Table 5:** Standardized and Unstandardized coefficients with the *p*-values of the MLR analysis.

Variable	Unstandardized Coefficients		Standardized Coefficients Beta	t	*p*-Value
	Coefficient	Std. Error			
Intercept	3.441	2.003		1.718	0.087
Age	0.106	0.029	0.107	3.623	**<0.001**
Gender	−0.018	0.677	−0.001	−0.027	0.978
Pre-operative LOS	1.009	0.035	0.809	28.558	**<0.001**
AMI	−1.773	1.812	−0.027	−0.979	0.328
CHF	2.548	0.728	0.100	3.502	**0.001**
PVD	4.586	2.027	0.065	2.262	**0.024**
CeVD	1.349	1.665	0.023	0.810	0.418
COPD	−0.536	1.310	−0.012	−0.410	0.682
Diabetes	−1.210	1.649	−0.021	−0.734	0.464
RD	0.552	1.309	0.012	0.422	0.673
2 Procedures	−0.358	0.767	−0.015	−0.466	0.642
3 Procedures	2.002	1.520	0.048	1.317	0.189
4 Procedures	0.166	2.513	0.002	0.066	0.947

**Table 6:** Evaluation metrics for the regression analysis.

	RF	NNN	Linear SVM	Rational Quadratic GPR
R^2^	0.670	0.580	0.690	0.710
Root Mean Squared Error	6.819	7.648	6.545	6.390

## Data Availability

The datasets generated and/or analyzed during the current study are not publicly available for privacy reasons but are available from the corresponding author on reasonable request.

## List of Abbreviations

LOS	Length of stay
MLR	Multiple linear regression
AMI	Acute myocardial infarction (AMI)
CHF	Congestive heart failure
CeVD	Cerebrovascular disease
PVD	Peripheral vascular disease
COPD	Chronic obstructive pulmonary disease
RD	Renal disease
RF	Random forest
SVM	Support vector machine
NNN	Narrow neural network
GPR	Gaussian process regression
